# Alternative splicing categorizes organ development by stage and reveals unique human splicing variants linked to neuromuscular disorders

**DOI:** 10.1016/j.jbc.2025.108542

**Published:** 2025-04-25

**Authors:** Chen Li, Fu-xing Gong, Zhigang Yang, Xin Fu, Hang Shi, Xuejian Sun, Xiaorong Zhang, Ran Xiao

**Affiliations:** 1 Research Center of Plastic Surgery Hospital, CAMS Key Laboratory of Tissue and Organ Regeneration, Chinese Academy of Medical Sciences and Peking Union Medical College, Beijing, China; 2 State Key Laboratory of Experimental Hematology, National Clinical Research Center for Blood Diseases, Haihe Laboratory of Cell Ecosystem, CAMS Key Laboratory for Prevention and Control of Hematological Disease Treatment Related Infection, Institute of Hematology & Blood Diseases Hospital, Chinese Academy of Medical Sciences & Peking Union Medical College, Tianjin, China

**Keywords:** alternative splicing, organ development, species variation, neuromuscular disorders

## Abstract

Alternative splicing (AS) diversifies protein expression and contributes to species-specific differences in organ development. Here, we focused on stage-specific splicing variants and their correlation with disease in humans compared to mice during brain and heart development. Temporal transcriptomic analysis revealed that splicing factors (SFs) can accurately classify organ developmental stages, and 5 SFs were identified specifically upregulated in humans during organogenesis. Additionally, inter-stage splicing variations were identified across analogous human and mouse developmental stages. Developmentally dynamic alternative splicing genes (devASGs) were enriched in various neurodevelopmental disorders in both species, with the most significant changes observed in human newborn brain and 16 weeks post-conception heart. Intriguingly, diseases specifically enriched in humans were primarily associated with neuro-muscular dysfunction, and human-specific neuromuscular devASGs were linked to mannose glycosylation and ciliary motility. These findings highlight the significance of SFs and AS events in organogenesis and inform the selection of appropriate models for translational research.

Understanding the process of organ development is fundamental to advancing our knowledge of the physiological and pathological functions of organs (1, 2, 3). Although rodents are extensively utilized as model organisms for researching tissue and organ growth and development, it is essential to delve into the notable differences that are specific to human (4, 5). A study conducted by Margarida Cardoso-Moreira *et al.* revealed that 51% of 13,471 genes exhibited distinct expression trajectories during human organ development when comparing to mice, and more than 200 genes were involved in brain, heart, and liver disease (4). AS is a widespread post-transcriptional regulatory process by which multiple different mRNA transcripts are produced from a single gene through various splicing patterns, thereby increasing the diversity of proteins and the richness of phenotypic traits in vertebrate organs for shaping species-specific differences (6, 7, 8). A multitude of diseases, ranging from developmental disorders to tumors, have been demonstrated to be associate with mutations occurring in cis-regulatory elements within pre-mRNA, trans-acting SFs, or other components of the spliceosome, leading to dysregulated AS (9). SFs participate in the recognition of splicing sites and the assembly of the spliceosome by binding to specific splicing cis-elements on pre-mRNA, thus playing a key function in AS (10). Therefore, investigating interspecies differences in AS during organ development is crucial to bridge the gap between mouse models and human biology, ultimately enhancing our understanding of human development and disease progression.

A comprehensive study on the development of eight organs in multiple species has confirmed that AS variations exhibit higher magnitude between species than between organs (11, 12, 13). AS exerts regulatory functions in the intricate interplay of gene expression levels during organ development, especially in the context of brain and heart development (14, 15). In the brain, AS participates in the dynamic regulatory processes underlying neuronal maturation, including significant changes in the morphology and function of individual neurons, as well as the formation of synaptic connections between neurons, thereby influencing the construction of complex neural circuits (9). Meanwhile, aberrant splicing is associated with several neurodegenerative disorders (14, 15, 16). Taking the retention of exon 19 transcript of the *Rbfox1* gene as an example, this splicing error leads to an abnormal isoform with altered intracellular localization, resulting in the loss of neuronal function (17, 18). In heart development, selective AS regulatory mechanisms have been discovered to play a crucial role in responding to varying metabolic conditions after birth (19, 20). Considering the critical role of AS in cellular differentiation and lineage determination, as well as the acquisition and maintenance of tissue identity in brain and heart, a research wave has been sparked in therapeutic strategies targeting aberrant splicing events to cure related diseases (14, 21, 22). The use of splice-switching antisense oligonucleotides (SSOs) to target splicing defects has shown significant therapeutic efficacy in treating spinal muscular atrophy (SMA) and Duchenne muscular dystrophy (23, 24, 25). The research into species differences in organ development could offer insights into the selection of animal models in drug research, tailored to species-specific considerations.

To elucidate the differences in alternative splicing between human and mouse during organ development, we conducted a focused analysis on the brain and heart. Utilizing publicly available transcriptome data (26), we analyzed gene expression time series to delineate the developmental phases of these organs. We systematically extracted the expression profiles of dynamically expressed genes and SFs, highlighting the pivotal role of SFs in developmental transitions. By integrating clustering of expression patterns with comparative inter-species analysis, we pinpointed SFs that exhibited a human-specific increasing trend after birth. Subsequently, we honed in on genes displaying splicing variations throughout development and discovered a human-specific enrichment in neuromuscular diseases. Through correlation analysis between selected SFs with neuromuscular disease associated devASGs, along with data from *HNRNPH2* knockout experiments, we validated the potential regulatory role of these SFs in human specific splicing variations. Furthermore, the integration of short-read and long-read RNA sequencing data provided robust evidence for these species-specific splicing changes. These findings prompted further exploration into the functional pathways and disease mechanisms associated with these splicing events during organ development.

## Results

### Expression patterns of splicing factors correspond with DDGs stage classifications and enable precise clustering during human organ development

**Figure 1:**
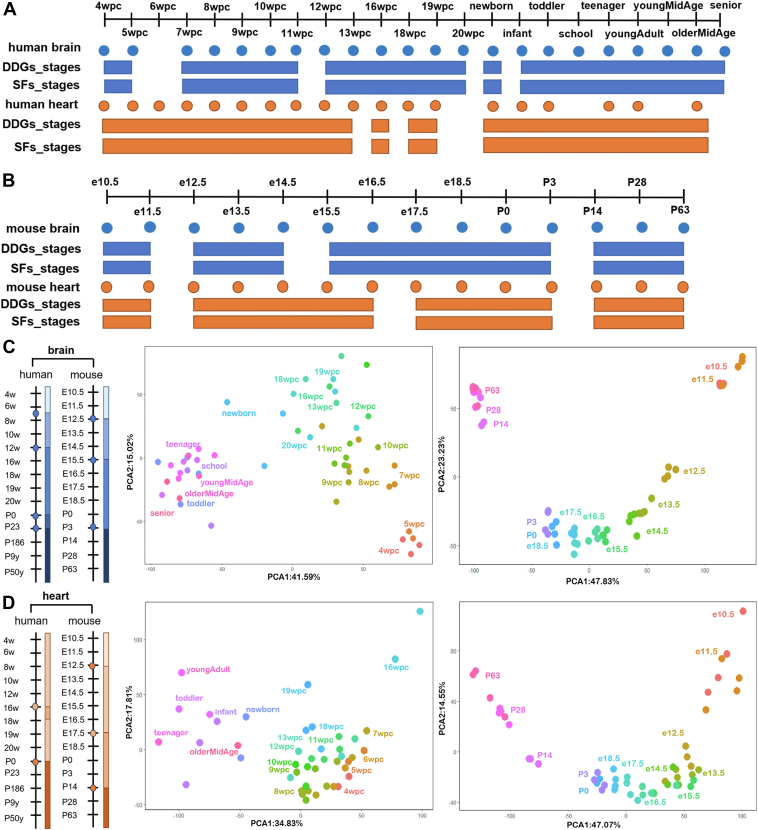
**Stage classification of human and mouse organ development.***A* and *B*, human and mouse tissue sampling and development stages. Dot points indicate the sampling time points, and the bars represent the classified developmental stages. *Blue*: Brain; *Orange*: Heart. DDGs_stages: Stage classification by DDG expression data; SFs_stages: Stage classification by SF expression data. *C* and *D*, *Left*: Stage correspondences across species during brain and heart development. *Middle*: Principal component analysis (PCA) of DDGs expression patterns during human development. *Right*: PCA of DDGs expression patterns during mouse development. The same color represents the same developmental stage in replicates. See also Figs. S1–S3.

To investigate the differential developmental processes of human and mouse, we collated the uploaded data in the previous literature (26), and analyzed developmentally dynamic genes (DDGs) and SFs of the brain and heart. For humans, the time series starts at 4 weeks post-conception (wpc) and continues throughout adulthood. The matching datasets of mouse are from 10.5 days post-conception (dpc) and then cover prenatal development daily until 18.5dpc as well as containing samples collected at postnatal day 0 (P0), P3, P14, P28, and P63. (Fig. 1, *A* and *B*).

Through hierarchical clustering of all genes at the transcriptome level, we identified distinct stage-specific distribution patterns of brain and heart in human and mouse (Figs. 1, *A* and *B*, S1, and S2). The results revealed that human brain development can be divided into five stages, with the unique clustering characteristics observed in the newborn stage, which was divergent from mouse brain development. In the context of heart development, human DDGs exhibited four distinct stages, with a notable expression pattern at 16 wpc, when the formation of cardiac chambers and the division of the atria and ventricles are completed, a process crucial for the heart’s functional maturation and adaptation. In comparison to human heart development, mouse heart appears to advance through three discernible stages prior to P3, indicating the species-specific differences in the temporal progression and features of these developmental landmarks. The same trend was confirmed by principal component analysis (PCA) (Fig. 1*C*). Furthermore, by conducting an expression correlation analysis across analogous developmental stages in both species, we identified robust correlations in brain development between the two species (Fig. S3); however, in the context of heart development, correspondence in stages was observed primarily between pre- and postnatal phases.

**Figure 2:**
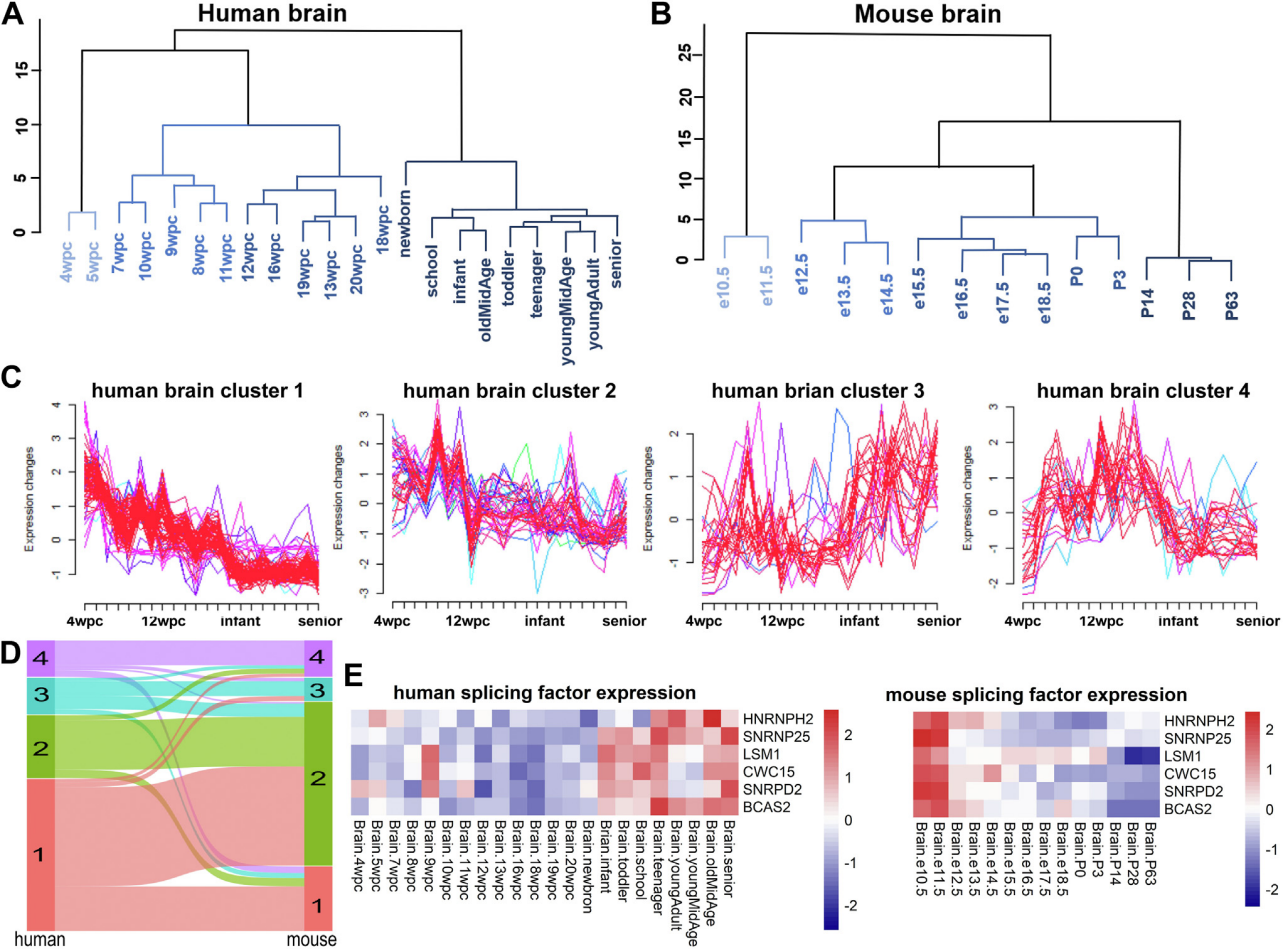
**Temporal profiles of splicing factors expression in human and mouse brain development.***A* and *B*, hierarchical clustering of SFs expression data during human and mouse brain development. *A*, human. *B*, mouse. *C*, fuzzy c-means clustering identified four distinct temporal patterns of SFs. The y axis shows the log normalized expression levels, and the x axis shows the samples ordered from early to late development. *D*, sankey diagram illustrating the comparative clustering of expression patterns for corresponding SFs between human and mouse. Each line represents a pair of human-mouse orthologs with a significant trajectory difference and shows the cluster assignment in human (*left*) and mouse (*right*). The lines are colored according to the human cluster assignment. *E*, Heatmap depicting SFs with species-specific differential expression trends during brain development. *Left*: Heatmap of species differential splicing factors in human. Right: Corresponding heatmap of expression trends for the same SFs in mouse. See also Fig. S4.

**Figure 3:**
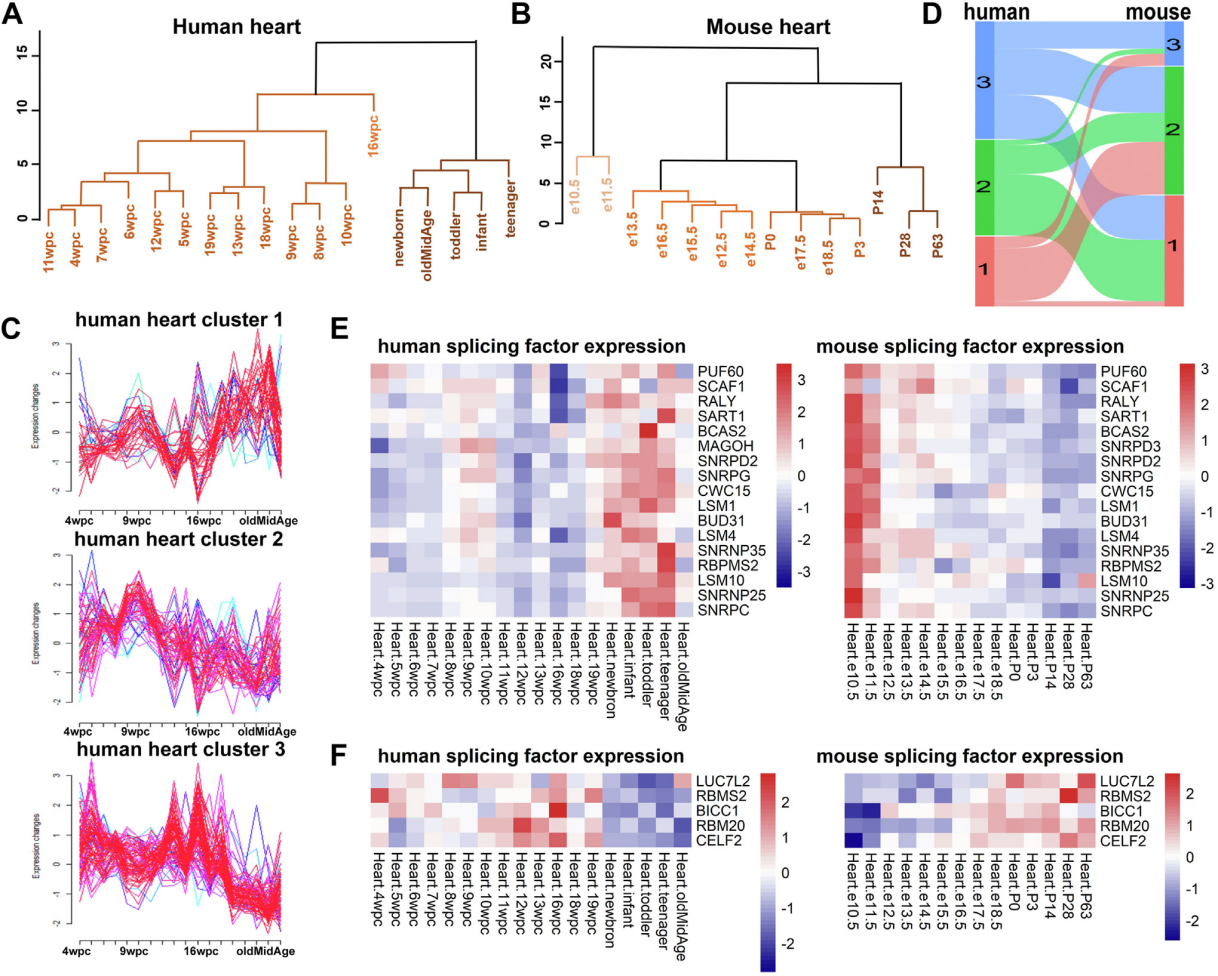
**Temporal profiles of splicing factor expression in human and mouse during heart development.***A* and *B*, hierarchical clustering of SFs expression data during human heart development. *A*, mouse. *B*, Human. *C*, fuzzy c-means clustering results of SFs expression trend during human heart development. The y axis shows the log normalized expression levels, and the x axis shows the samples ordered from early to late development. *D*, sankey diagram show the clustering comparison of expression patterns for the same SFs in human and mouse. Each line represents a pair of human-mouse orthologs with a significant trajectory difference and shows the cluster assignment in human (*left*) and mouse (*right*). The lines are colored according to the human cluster assignment. *E* and *F*, Heatmap of SFs with species-specific expression trends during heart development. *Left*: SF expression trends in human. *Right*: SF expression trends in mouse. *E*, SFs upregulated during the late-stages of human heart development but downregulated in mouse. *F*, SFs downregulated during the late-stages of human heart development but upregulated in mouse. See also Fig. S5.

Recognizing the pivotal function of alternative splicing in organ development (27, 28, 28), we extracted the expression matrix of SFs and found that its hierarchical clustering in both human and mouse corresponded with the developmental stage classifications derived from DDGs profiles (Fig. 1, *A* and *B*). Notably, when applied to human organ development, there were a few nuanced differences in the clustering patterns between the two methods (Figs. 2*A*, 3*A*, S1, and S2). For instance, the newborn brain's expression pattern was more closely related to postnatal stages in the SFs-based classification; however, it shows a stronger relation to prenatal stages of the 12wpc to 20wpc period in the DDGs-based classification. Similarly, in the SFs clustering, the 13wpc heart sample was grouped more with the 18wpc to 19wpc subcluster, while it was more closely associated with the 8wpc to 10wpc subcluster in the DDGs classification. These distinctions illustrate the precedence of SFs’ expression in modulating gene expression throughout the developmental process of human organs.

### The majority of splicing factors peak in expression during early development, with a distinct human-specific cluster exhibiting a postnatal increase in brain

In the unsupervised hierarchical clustering analysis of brain SFs expression profiles, the human newborn stage showed a significant shift of gene expression profile (Fig. 2*A* and Tables S1, and S2). However, in mice, SFs expression did not immediately mirror the postnatal stage phenotype until P14 (Fig. 2*B*). We further applied the fuzzy c-means algorithm to cluster SFs expression patterns, and SFs expression kinetics of both human and mouse were represented by four distinct clusters of temporal patterns (Fig. S4). The majority of SFs in the brain of both species exhibited a higher expression during the early developmental stages and a gradual decline in the later stages, which aligns with the organ pluripotency at the early stages reported in previous studies (9). However, the expression of SFs in human cluster 3 increased after birth and corresponded to all four clusters in mouse (Fig. 2, *C* and *D*).

In fact, about one-third of SFs in human cluster 3 were also observed in mouse cluster 2, whose expression exhibited an opposite pattern during mouse brain development. Differential expression patterns of 6 SFs overlapped in human cluster 3 and mouse cluster 2 were displayed by heatmap, showing increased expression during the later stages of human brain development and a gradual decrease in expression during mouse brain development (Fig. 2*E*). Among them, *HNRNPH2* and *LSM1* have been implicated to be associated with various neurodevelopmental disorders in numerous studies (29, 30, 31). Moreover, the species-specific expression of these SFs during different stages of H9-derived forebrain neuron differentiation and mouse brain development was confirmed by qPCR (Fig. S5). These results demonstrated a specific temporal expression pattern of human brain SFs after birth, suggesting their involvement in a complex and ongoing process of postnatal brain development.

### The expression patterns of splicing factors in the heart exhibit significant species-specific differences, with a conserved postnatal upregulation pattern in human

As the first organ to form and function during the embryonic development stage (32), the heart displays unique expression patterns among its SFs (Fig. 3, *A* and *B*, Tables S1, and S2). SFs' temporal expression patterns of both human and mouse were represented by 3 distinct clusters (Figs. 3, *C* and *D*, and S6). One-fourth of SFs were divided into Cluster 1 of the human heart, with an increasing expression tendency after birth (Fig. 3*C*). On the contrary, SFs Clusters 2 and 3 of the human heart showed a decreasing temporal expression pattern. In the mouse heart, Clusters 1 and 2 displayed gradually decreasing expression patterns during later developmental stages, whereas the expression pattern of Cluster 3 exhibited an increasing trend (Fig. S6).

The analysis of Sankey diagrams disclosed a correlation between the distinct temporal expression patterns in heart development across human and mouse (Fig. 3*D*). SFs overlapped in human Cluster 1 and mouse Clusters 1 and 2 were selected and illustrated by heatmap, showing increased expression during the later stages of human heart development and a gradual decreased expression during mouse heart development (Fig. 3*E*). Many of these SFs have been implicated in human cardiac-related cases, with reports of mutations or functional abnormalities. It is worth noting that heterozygote loss-of-function variants in *PUF60* has been associated with atrial septal defects and the development of aortic arch abnormalities in multiple case reports (33). Additionally, there are SFs exhibiting increased expression during the later stages in mice but the opposite expression trend in human heart development (Fig. 3*F*). *RBM20* is extensively studied and regarded as a regulator in the process of sarcoplasmic reticulum Ca^2+^ release in severe arrhythmogenic dilated cardiomyopathy (34). The interspecies differences in these SFs may lead to specific regulation for morphology and function during heart development. Moreover, in the postnatal development of both the brain and heart in human, we observed species-specific upregulation of *SNRNP25*, *SNRPD2*, *LSM1*, *CWC15* and *BCAS2* (Figs. 2*E* and 3*E*), suggesting a conserved regulatory pattern that is unique to human and plays a role in the physiological maturation of these vital organs.

### The developmentally dynamic splicing genes in the human brain and heart are species specifically enriched in multiple neuromuscular developmental diseases

**Figure 4:**
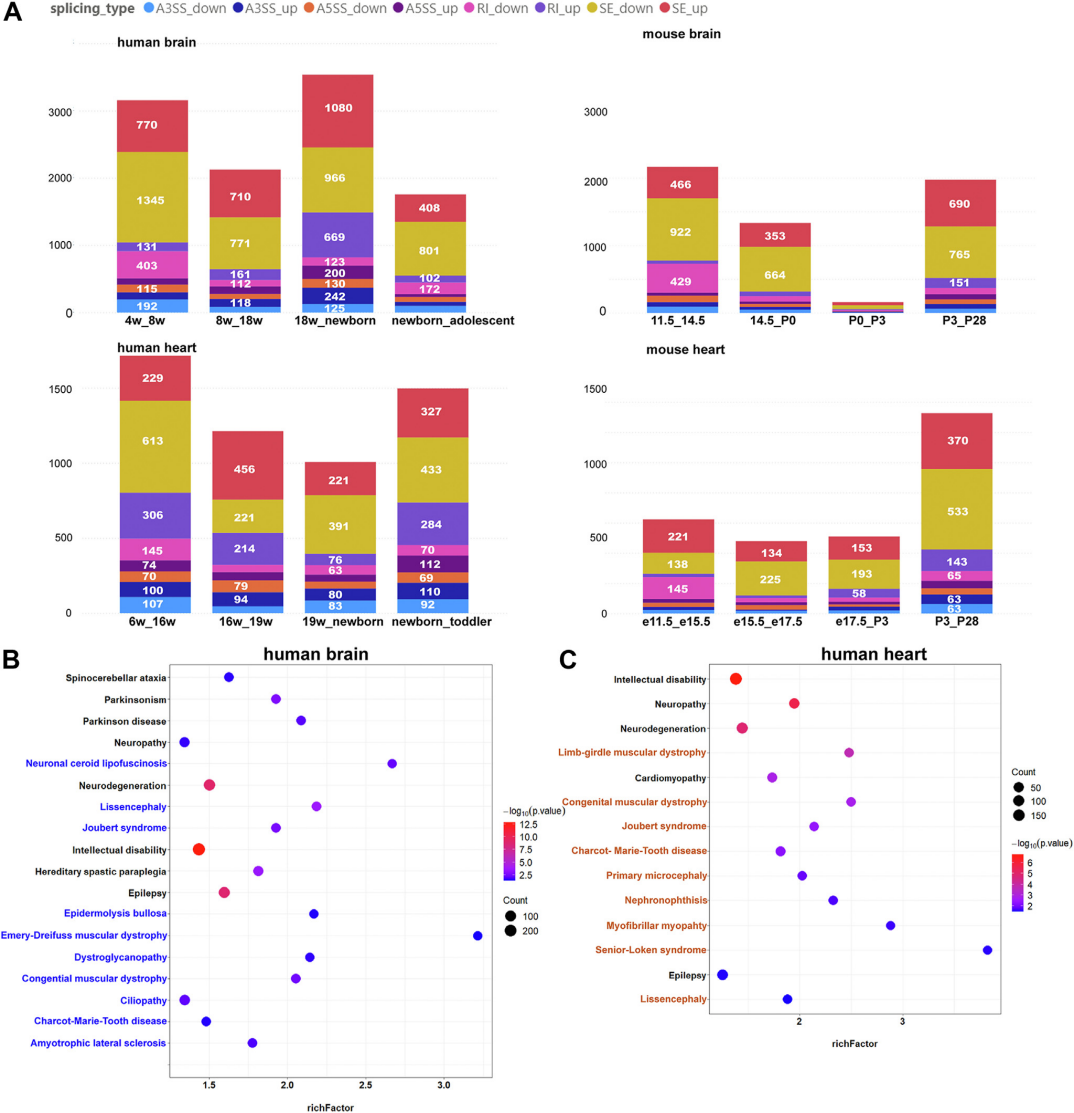
**Splicing events in critical developmental stages and diseases enrichment of devASGs.***A*, number of differential splicing events across developmental stages defined by DDGs and SFs expression profiles during brain and heart development in human and mouse. *B*, diseases enriched by devASGs during human brain development. The highlighted diseases in *blue* are specifically enriched during human brain development compared to mouse. *C*, diseases enriched by devASGs during human heart development. The highlighted diseases in *orange* are specifically enriched during human heart development compared to mouse. A3SS, Alternative 3′ splice site; A5SS, Alternative 5′ splice site; RI, retained intron; SE, skipped exon. See also Figs. S6–S8.

To further investigate the splicing events during the development of brain and heart, we used rMATS to analyze different types of alternative splicing events, including skipped exon (SE), retained intron (RI), mutually exclusive exon (MXE), alternative 3′ splice site (A3SS), and alternative 5' splice site (A5SS), and compared inter-stage splicing events based on the stage-specific classifications and the analogous developmental stages in both species. The results indicated that there were more devASGs in human than in mouse, and SE was the most prominent category of alternative splicing events during the development of brain and heart in both human and mouse at various stages (Fig. 4*A* and Table S3). During human development, a significant surge in alternative splicing events was observed in the brain from the 18wpc to the newborn stage, characterized by a notable increase in the SE_up and RI_up indexes. Similarly, in the heart, a pronounced enhancement in splicing events was detected from the 6wpc to the 16wpc, marked by a significant elevation in the SE_down and RI_down indexes. The observation confirmed the distinct classification of developmental stages at birth for the brain and 16wpc for the heart in humans. However, the mouse heart showed a significant rise in splicing events from P3 to P28, contrasting with the relative stability of splicing patterns observed during embryonic development. Interestingly, the shared devASGs splicing events during postnatal stages in human and mouse are mainly involved in cytoskeleton organization and synaptic signaling in the brain, as well as ion transmembrane transport and muscle contraction in the heart (Fig. S7, *C*–*E*).

We then assigned devASGs in human and mouse organogenesis to explore the potential correlation between the spatiotemporal splicing changes and phenotypic manifestation of human diseases based on the UniProt UP_KW_DISEASE database. Within the brain development, devASGs were enriched in a variety of neurodevelopmental disorders, including intellectual disability, neurodegeneration, epilepsy, Parkinson's disease, and so on, in both human and mouse (Figs. 4*B*, S7*A*, and Tables S4–S7). As for the heart development, cardiomyopathy showed organ-specific enrichment in both human and mouse (Figs. 4*C*, S7*B*, and Tables S4–S7). These devASG expression not only varies between species at equivalent developmental stages but also occurs at different stages within each species (Fig. S8).

Moreover, neurological disorders were significantly enriched by devASGs in both the brain and heart. This aligns with the observation that genes causing diseases of the nervous system tend to be ubiquitously expressed, while those impacting the heart are typically more localized (4). In this study, we observed distinct splicing changes in Exon 8 of the *APP* gene, which has been implicated in neurodegenerative diseases (35). These splicing patterns exhibited notable differences between brain and heart development at each developmental stage (Fig. S9). Intriguingly, a majority of the neuromuscular disorders were specifically enriched during human brain and heart development, including Joubert syndrome, congenital muscular dystrophy, neuronal ceroid lipofuscinosis, amyotrophic lateral sclerosis (ALS), dystroglycanopathy, Charcot-Marie-Tooth disease, and Emery-Dreifuss muscular dystrophy (Figs. 4*B* and S7). This finding indicates a specific association between human devASGs and the pathogenesis of neuromuscular diseases.

### Human-specific neuromuscular devASGs are predominantly associated with human-specific splicing factors and are critically involved in the processes of mannose glycosylation and ciliary motility

**Figure 5:**
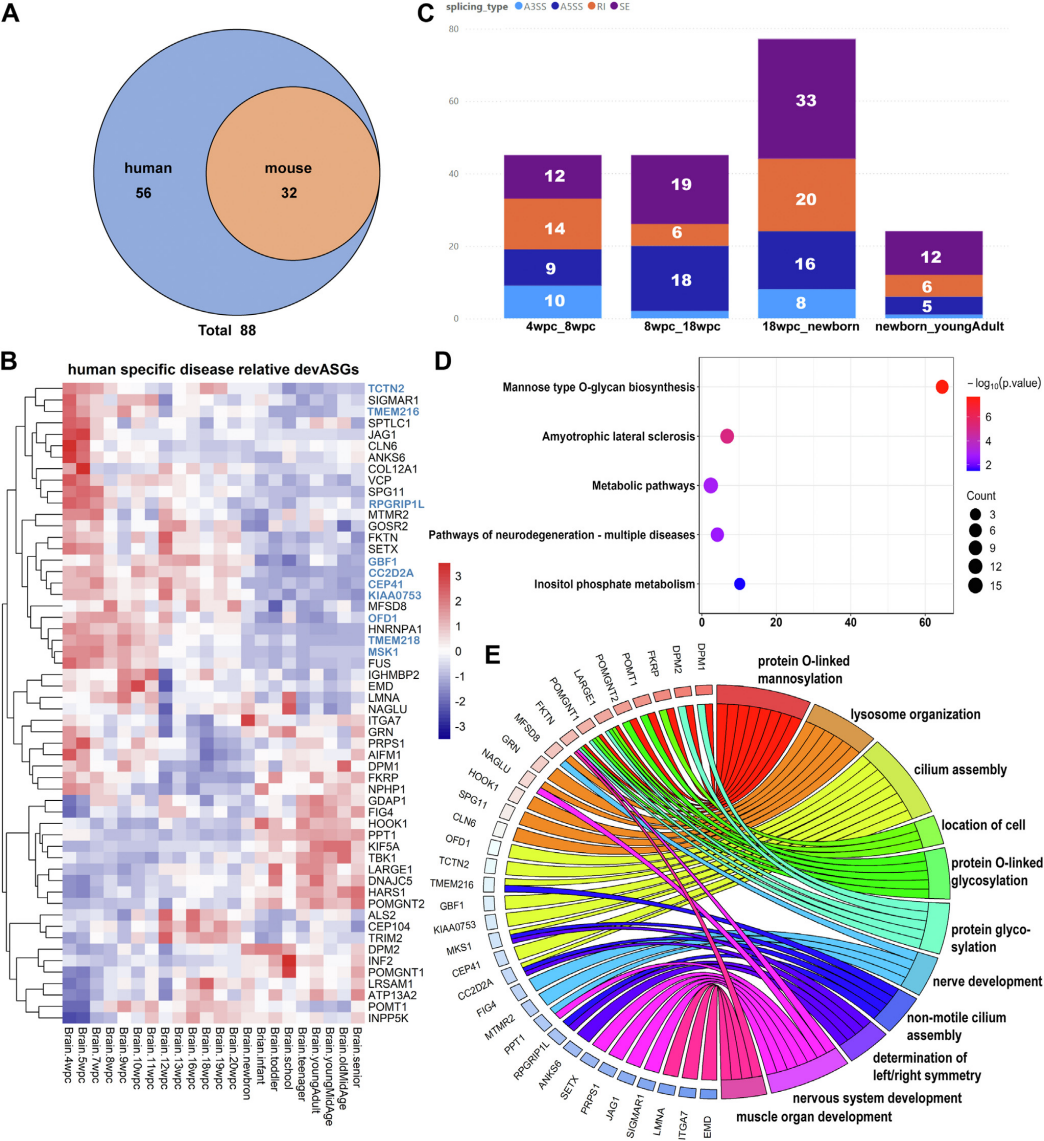
**Temporal expression and functional analysis of human-specific devASGs associated with neuro-muscular disorders.***A*, identification of human-specific devASGs associated with neuromuscular disorders. *B*, temporal expression of human-specific devASGs. *C*, number of splicing events in human-specific devASGs across brain developmental stages. *D*, KEGG pathway cluster analysis of human-specific devASGs. *E*, Chord plot illustrating 11 significantly clustered GO biological process pathways of human-specific devASGs and genes involved in these pathways.

We then selected devASGs associated with neuro-muscular diseases, and 56 human-specific devASGs in the brain were identified by excluding those present in mouse (Fig. 5*A* and Table S8). Analysis of their temporal expression patterns revealed that the majority of these devASGs were significantly expressed either during early developmental stages or at later stages (Fig. 5*B*). Their splicing events during human brain development exhibited the highest variability at the transition to birth (Fig. 5*C*). Moreover, KEGG and GO functional clustering analyses showed these human-specific devASGs were mainly enriched in protein O-linked mannosylation, lysosomal dysfunction, cilium assembly, nerve development, and muscle organ development (Fig. 5, *D* and *E*). Notably, the majority of cilia assembly-related devASGs (marked by blue color in Fig. 5*B*), whose abnormal expression is implicated in Joubert syndrome (36), exhibited significant upregulation during the early developmental stages in the human brain. This pattern of expression suggested a potential early time point for the onset of molecular pathology underlying Joubert syndrome.

**Figure 6:**
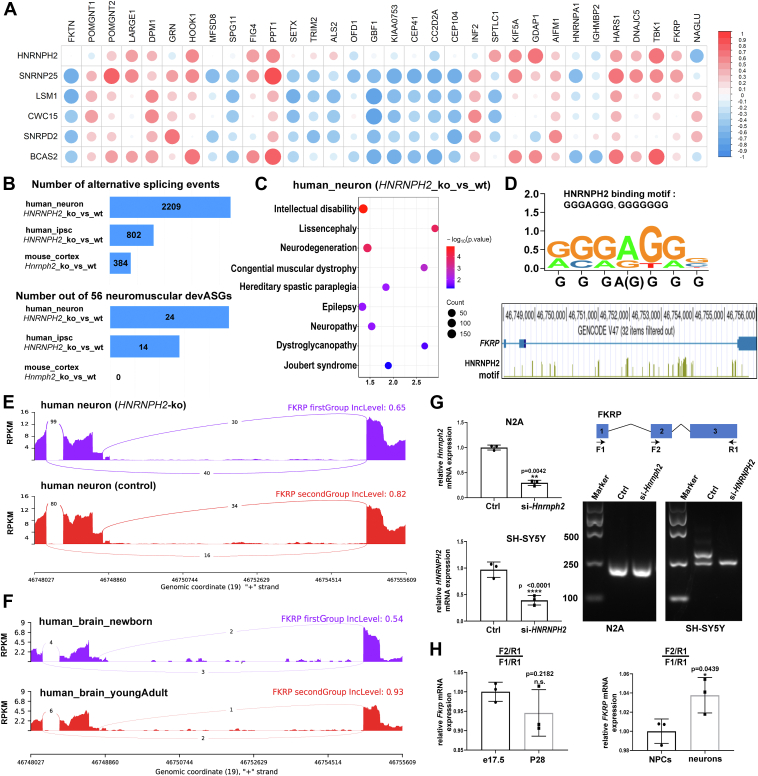
**Transcriptional correlations between human-specific SFs and neuro-muscular devASGs**. *A*, transcriptional correlation between human-specific SFs and neuro-muscular devASGs during brain development. The color intensity of the data points corresponds to the magnitude of the correlation coefficient. *B*, number of alternative splicing events and neuromuscular devASGs induced by *HNRNPH2* knockout in iPSC/iPSC-derived neurons and mouse cortex. *C*, diseases enriched by *HNRNPH2* knockout induced splicing events in human iPSC-derived neuron. *D*, *Top*: Multiple sequence alignment of 24 neuromuscular devASGs fragments with splicing variations; *Bottom*: RBPmap motif analysis highlighting 32 potential HNRNPH2 binding sites near the *FKRP* splicing variation sequences. *E*, *FKRP* splicing change induced by *HNRNPH2* knockout in human iPSC-derived neurons. *F*, *FKRP* splicing patterns in human brain at newborn and youngAdult stages. *G*, *FKRP* splicing analysis using F1/R1 primers in human SH-SY5Y and mouse N2A cell lines after knockdown of *HNRNPH2*. *H*, *FKRP* splicing analysis using F1/R1 primers in human H9-derived NPCs, neurons, and brain tissues from e17.5 and P28 mice. Unpaired Student's *t* test, Mean ± SD, n = 3 biological replicates, with technical duplicates; ∗*p* < 0.05, ∗∗*p* < 0.01, ∗∗∗∗*p* < 0.0.0001.

Therefore, to uncover possible regulatory effects of 6 human-specific upregulated SFs on the 56 human-specific devASGs during brain development, we conducted a further investigation into their transcriptional correlations. The results showed that 32 devASGs demonstrated a correlation coefficient exceeding 0.5 with at least one SF (Fig. 6*A*), indicating a significant transcriptional relationship. Specifically, 8 devASGs showed a higher correlation coefficient greater than 0.6 with SNRNP25, and 7 devASGs with BCAS2, suggesting that these two splicing factors play a pivotal role in guiding the temporal regulation of human-specific molecular dynamics associated with neuromuscular disorders. Additionally, five human-specific upregulated splicing factors, *SNRNP25*, *LSM1*, *CWC15*, *SNRPD2*, and *BCAS2*, displayed a similar pattern of correlation with a set of devASGs associated with cilia function, including *GBF1*, *KIAA0753*, *CEP41*, *CC2D2A*, and *CEP104*.

Given that HNRNPH2 is one of the most extensively studied splicing factors, we subsequently integrated RNA-seq data from *HNRNPH2*-knockout human iPSCs/iPSC-derived neurons and *Hnrnph2*-knockout mouse cortex (37). We quantified the number of altered splicing events and human-specific devASGs associated with neuromuscular diseases. The results indicated that HNRNPH2 had the most significant impact on splicing events in human iPSC-derived neurons compared to human iPSCs and mouse cortex (Fig. 6*B*). Moreover, the splicing changes induced by *HNRNPH2* knockout in human neurons were predominantly enriched in genes associated with neuromuscular diseases (Fig. 6*C*). Among the 56 human-specific neuromuscular devASGs that we previously identified, 24 exhibited splicing changes in *HNRNPH2*-knockout human neurons. We then performed multiple sequence alignment of splicing sites in 24 devASGs and identified a consistent HNRNPH2 binding motif (Fig. 6*D*). We focused particularly on FKRP (fukutin-related protein), a Golgi glycosyltransferase that encodes an enzyme essential for the glycosylation of α-dihydroglycolipids (α-DG). FKRP plays a crucial role in the development of the muscle and nervous systems, primarily in stabilizing and maintaining the function of muscle cell membranes (38). Sashimiplot revealed that *HNRNPH2* knockout led to increased exon 2 skipping in *FKRP*, a change also observed in human newborn brain compared to young adult brain (Fig. 6, *E* and *F*). Furthermore, we validated the splicing variants of *FKRP* using primers targeting different exonic regions in the human glioblastoma cell line SH-SY5Y and the corresponding mouse cell line N2A. We found that *HNRNPH2* knockdown altered the splicing pattern of *FKRP* only in SH-SY5Y cells, where exon 2 inclusion was reduced (Fig. 6*G*). The human-specific increase in FKRP exon 2 inclusion during late-stage neuronal development was then confirmed by comparing its splicing alterations in H9-derived NPCs and forebrain neurons with brain tissues from e17.5 and P28 mice (Fig. 6*H*). Altogether, these findings demonstrate that HNRNPH2 plays a stage-dependent and species-specific role in human brain development by regulating the alternative splicing of downstream devASGs.

### Long-read sequencing data validate the splicing variants of human-specific neuromuscular devASGs

Long-read sequencing has emerged as an ideal tool for elucidating the AS landscape and distinguishing splicing changes across different groups. In our study, we utilized a comprehensive dataset spanning developmental stages in both human and mouse samples. This dataset is particularly valuable, given the difficulty in obtaining tissues that cover the entire human developmental timeline. To address the limitations of short RNA sequencing reads in identifying isoforms with complex AS events, we searched publicly available databases covering human and mouse brain developmental processes and validated our results using long-read sequencing data from two datasets from developing human brain and mouse neural stem cells (NSCs) (39, 40).

**Figure 7:**
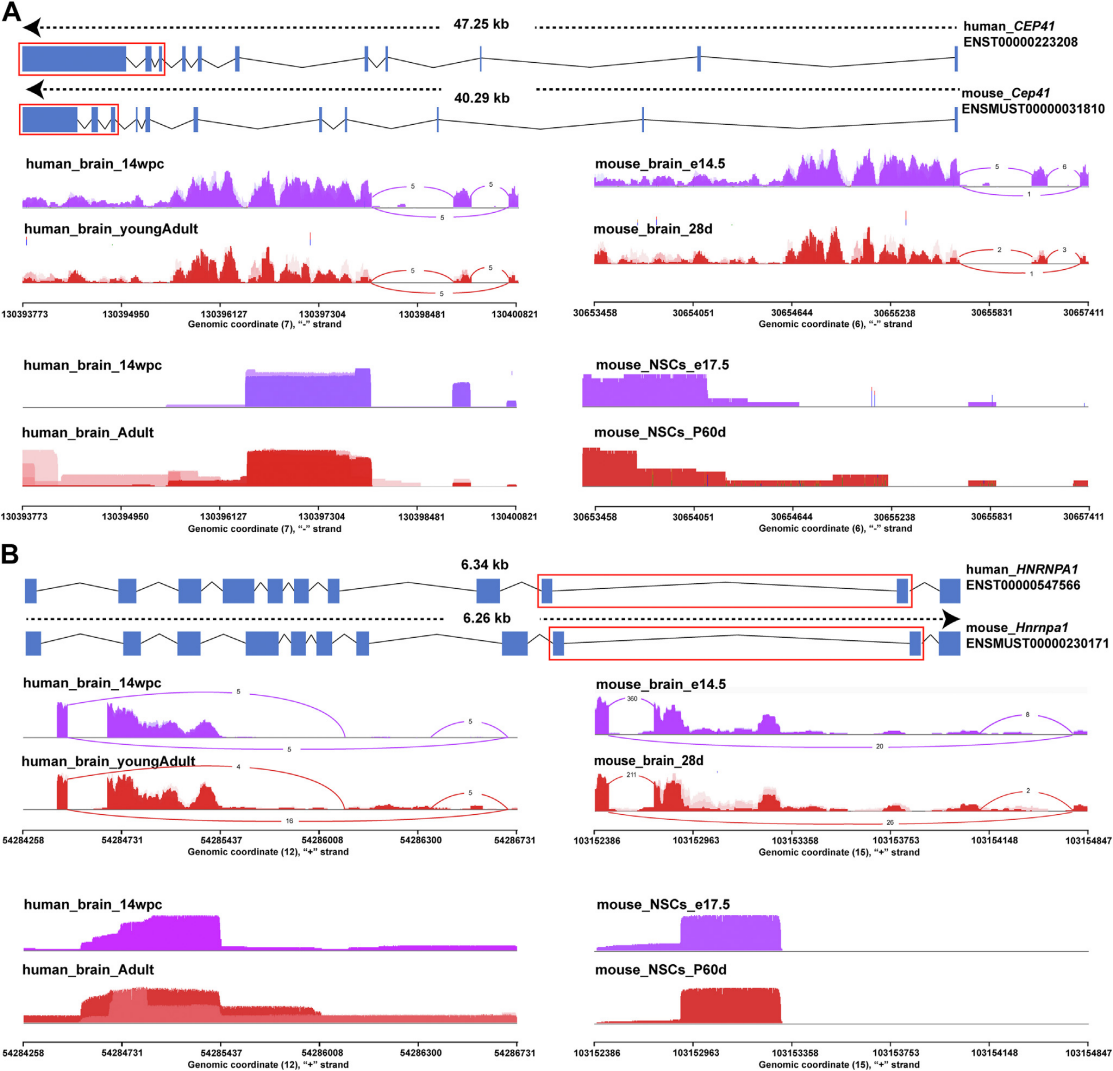
**Splicing patterns of neuro-muscular devASGs in human and mouse**. *A* and *B*, *top*: Differential splicing regions in gene structure schematic are highlighted in *red* box. *A*, *middle*: Splicing patterns of *CEP41* based on short-read sequencing data. *Bottom*: Splicing patterns of *CEP41* based on long-read sequencing data. *B*, *middle*: Splicing patterns of *HNRNPA1* based on short-read sequencing data. *Bottom*: Splicing patterns of *HNRNPA1* based on long-read sequencing data.

For example, *CEP41*, a gene involved in ciliary glutamylation and essential for axonemal formation, displayed exon 10 SE splicing in humans during the transition from embryonic to postnatal stages, whereas mice consistently expressed exon 10 throughout brain development (Fig. 7*A*). The inclusion of exon 10 induces a structural shift in the core domain of CEP41, transitioning from the Rhodanese_3 to the RHOD_4 conformation (Fig. S10*A*). This domain is critical for microtubule binding, assembly, and the regulation of cell division and the cell cycle (41). Notably, mutations in *CEP41* that disrupt splice site selection and protein function have been linked to Joubert syndrome (42). Another example is *HNRNPA1*, which exhibits human-specific splicing events that alter protein length from newborn to young adult stages (Fig. 7*B*). This splicing variation occurs within a low-complexity domain, a region where pathogenic mutations in neuromuscular diseases are frequently concentrated (43, 44). Altered expression in this region has been shown to impact the stability of the steric zipper motif, potentially leading to dysregulation of protein aggregation. We further investigated additional splicing changes in human-specific neuromuscular devASGs, consistently identifying species-specific differences at both short-read and long-read sequencing levels (Fig. S10, *B*–*D*).

Overall, our findings highlight the importance of human-specific splicing factors and their regulation of neuromuscular developmentally associated splicing genes. These splicing patterns during critical developmental stages provide key insights into the molecular pathology underlying neuromuscular diseases in the context of human brain development.

## Discussion

The species-specific differences in SFs and AS events during organ development, along with the potential implications of these differences in the context of various diseases, have been largely neglected in previous literature. This study aimed to address the significant gap by examining the temporal expression of SFs and human-specific splicing events during the development of the brain and heart, in contrast to their expression in the mouse. SFs are integral to the splicing machinery, playing a pivotal role in recognizing binding sequences and facilitating the assembly of pre-mRNA (45, 46). Here we discovered that the temporal dynamics of the SFs expression profiles were intricately aligned with the developmental stages of organs, which synchronization may provide a new criterion for classifying organ phenotypes by stage and understanding species-specific differences. This underscores the influence of SFs on shaping the transcriptome and regulating gene expression during organ development.

Moreover, the distinct expression patterns of SFs between humans and mice provide insights into the unique splicing programs that drive development in each species. Notably, several SFs, such as *SNRNP25*, *LSM1*, *CWC15*, *SNRPD2*, and *BCAS2*, exhibit a species-specific increase in expression during the postnatal stages of human brain and heart development. *SNRNP25* is involved in the assembly and the splicing process for accurate RNA splicing (47). LSM1 contributes by forming a ring complex that influences mRNA degradation and RNA metabolic regulation, thereby affecting splicing efficiency (31). CWC15, SNRPD2, and BCAS2 are integral to the spliceosome's assembly and function, with CWC15 being a subunit of the Prp19/CDC5 complex (48), SNRPD2 a component of the Sm ring (49), and BCAS2 a core component of the Prp19 complex (50, 51), all crucial for the catalytic activation of the spliceosome. We also established a correlation between human-specific SFs and neuromuscular devASGs, highlighting their crucial roles in pathological mechanisms of neuromuscular diseases. How they contribute to the fine-tuned regulation in human postnatal development in a species-specific manner needs to be elucidated.

Recent studies suggest that around one-third of disease-causing mutations are linked to RNA splicing (21, 52, 53). Interestingly, we identified a distinct pattern in neuro-muscular diseases specifically enriched in humans, which correlated with differential splicing events at critical developmental stages. Our research revealed that notable interspecies disparities in the dynamics of alternative splicing were within genes associated with neuromuscular diseases. The processes of mannose glycosylation and ciliary motility were linked to human-specific neuromuscular devASGs. O-mannose glycosylation, a cell surface receptor modification, is essential for maintaining cell membrane integrity in regulating muscle function and plays a role in peripheral myelination, neuromuscular junction formation, and neuronal migration in the brain (23). Primary cilia are microtubule-based organelles that project from the cell surface and play crucial roles in neural tube development and synapse formation between pyramidal neurons of the hippocampal CA1 region and axons of brainstem serotonergic neurons (54, 55). Although the signaling pathways that govern cilia organization are evolutionarily conserved, there are distinct differences in the implementation of the Hedgehog and Planar Cell Polarity pathways between fruit flies and mammals (56). These insights deepen our understanding of the molecular underpinnings of neuromuscular diseases and emphasize the potential contribution of species-specific splicing patterns for distinct manifestations in humans.

During development, splicing events at various stages generate proteins that are specifically tailored to meet the organism's temporal needs. Our data showed a significant surge in alternative splicing events from the 18wpc to the newborn stage in the human brain and from the 6wpc to the 16wpc in the human heart, concordance with two specific developmental stages classified by SFs expression profiles in humans. Colantuoni *et al.* found waves of gene expression changes that occur during fetal development and are reversed in early postnatal life in the prefrontal cortex (57, 58). Comparative analysis of splicing events between fetal and adult brains revealed that a mere 24% of these changes overlap, indicating that a substantial number of splicing events are developmentally stage-specific (59, 60). The fetus at 16 weeks of gestation has entered a period of rapid growth with functional maturation of the heart's conduction system (20, 61, 62). Moreover, we observed downregulated SE events in the human brain after birth, suggesting that postnatal proteins tend to have more amino acids. A study by Sebastie M.W. *et al.* reported similar findings in mouse cortical development, where a reduction in SE led to an enrichment of *LSD1*, enhancing neuronal phosphorylation sites and promoting axon growth (9, 63). Therefore, it is hypothesized that the increase in peptide segments may augment neuronal plasticity in brain cells postnatally, facilitating adaptation to the environment. This discovery provides valuable insights into splicing regulation during a critical period of development.

Multiple studies have revealed phenotypic discrepancies between rodent models of ALS and SMA and human patients. These differences in disease manifestations are largely attributed to splicing variations in genes such as *UNC13 A* and *SMN*, where the pathogenic exons present in humans are absent in mice (64, 65, 66, 67). Understanding the regulatory mechanisms governing these splicing events is critical for advancing our knowledge of organ development and for designing targeted therapeutic strategies. Our findings on HNRNPH2-mediated modulation of exon 2 skipping in *FKRP* establish a causal relationship between human-specific SFs and neuromuscular devASGs. Future investigations could provide more valuable insights by elucidating the specific mechanisms driving interspecies splicing differences. This includes exploring the roles of key molecules such as HNRNPH2, SNRNP25, LSM1, CWC15, SNRPD2, and BCAS2 in disease pathogenesis, as well as examining differences in downstream splicing regulatory patterns between patients and their murine counterparts.

## Experimental procedures

### Data resource

Whole-stage tissue developmental raw and processed RNA-seq data we used were download in ArrayExpress by using the accession codes: E-MTAB-6798 (mouse), E-MTAB-6814 (human) (https://www.ebi.ac.uk/arrayexpress/) (26). We analyzed data from 203 strand-specific RNA-seq libraries sequenced: 52 from human brain, 43 from human heart, 54 from mouse brain, and 54 from mouse heart. The fastq data used for further analysis of splicing patterns are shown in Table S3. *HNRNPH2*-KO human iPSCs-derived neurons and mouse cortex RNAseq data were downloaded in GEO datasets by using the accession codes: GSE226525 (human) and GSE226526 (mouse) (37).

### DDGs selected and developmental stage analysis

We used the matrix of fragments per kilobase of transcript per Million mapped reads (FPKM) downloaded in ArrayExpress to select the genes with dynamic temporal profiles using maSigPro in each organ, which an R package designed for transcriptomic time courses (68, 69). After selecting DDGs, we employed PCA for dimensionality reduction and utilized its results to conduct hierarchical clustering to determine stage-specific clustering of organ development. Additionally, we integrated the results of the screen plot to determine the optimal number of clusters for classifying organ developmental stages.

### Stage correspondences across species in each organ

We concerted the gene names from mouse to human using the R package AnnotationDbi (v1.56.2) and constructed an expression matrix of the 1:1 DDGs in human and mouse for each organ’s developmental process and performed cross-species stage correspondence analysis. Then we calculated the Spearman correlations between all developmental time points in the brain and heart using ggcorrplot R package (v0.1.4.1) between human and mouse, and showed the correlation coefficient by dot plot.

### Analysis of splicing factors expression

A total of 221 human and 214 mouse genes were selected for analysis based on literature mining (70). To calculate the mRNA expression values for each sample, we used the value of FPKM to measure the expression level of each selected SF genes. And selected SFs with dynamic temporal profiles by maSigPro. By employing PCA analysis, we effectively reduced the dimensionality of our data, establishing a foundation for hierarchical clustering. This approach allowed us to delineate the developmental stages of each organ based on the distinct expression patterns of SFs. Besides, we used Mfuzz, a R software package for sort clustering of expression matrix (71), to categorize the SFs into different groups based on their expression patterns, with the specific number of categories determined by the inflection points in the scatter plot. The genes contained in each cluster were integrated, and their expression patterns were plotted as line graphs. Sankey diagrams were employed to compare the clustering of SFs expression patterns between mouse and human and to determine the correspondence of clusters between species. Additionally, by integrating the Sankey diagrams and line plots, we identified genes with distinct expression patterns during heart and brain development in human and mouse. The differential expression patterns were visualized using a heatmap implemented with the R package: pheatmap.

### Alternative splicing analysis

Then we analyzed the splicing gene according to the following four steps. In step 1, we used FastQC (v0.12.1) to assess the quality of omics data and determine further processing methods. In step 2, we removed adapter sequences and unstable sequencing reads identified during the quality control process by cutadapt (v 3.2). In step 3, all RNA-seq reads from our selected library for human and mouse were mapped to the corresponding genome sequences using TopHat2 (v 2.1.1) (72). Finally, replicate multivariate analysis of transcript splicing (rMATs, v4.0.2) was used to identify differentially spliced gene corresponding to five major types of AS patterns, including SE, MXE, A3′SS, A5′SS, and RI. Data were carried out according to the previous literature (73).

### Dynamic alternative splicing genes selected

Significant AS events were considered if average coverage >5 and delta percent spliced in (ΔPSI) > 0.1 (74). Using the stacked bar chart module in Power BI (v 2.128.1177.0), we conducted a statistical analysis of the alternative splicing changes between stages during human and mouse heart and brain development. Additionally, within the same species and organ, we integrated the alternative splicing genes across different stages and patterns to devASGs during the organ's developmental process. The human brain development stages yielded 4308 devASGs, while the mouse brain had 2708 devASGs. In terms of heart development, we identified 2830 devASGs in human and 1720 devASGs in mouse.

### Visualizations of splicing patterns

We used rmats2sashimiplot (v 3.0.0) to analyze the change of splicing pattern, code used to draw this plot is shown below: rmats2sashimiplot --b1 accepted_hits1.bam --b2 accepted_hits2.bam --event-type SE -e path/to/output/SE.MATS.JC.txt --l1 timepoint1 --l2 timepoint2 --exon_s 1 --intron_s 5 -o output --group-info timepoint1_timepoint2.gf/to/Mus_musculus.GRCm39.105.chr.gff3.

### Disease, GO, and KEGG enrichment analysis for devASGs

The enrichment analysis for the relative disease of devASGs was carried out by the web-based Database for Annotation, Visualization, and Integrated Discovery (DAVID) functional annotation tools. Functional clustering of devASGs relative to disease was selected based on the UniProt UP_KW_DISEASE database. Besides, we identified a category of movement-related diseases specific to human brain development and extracted the human-specific devASGs within these pathways for further functional analysis. To identify and compare the presence of alternative splicing events in neuro-muscular disease-associated devASGs between human and mouse, we constructed Venn diagrams and subsequently filtered out 56 devASGs exhibiting splicing variations exclusively during human brain development. The significance threshold for enriched GO terms was set at a *p*-value of 0.05, FDR correction. The results of enriched KEGG pathways were shown with their corresponding *p*-values, rich factors, and the number of genes in the input list associated with each KEGG term. We also used the R package GOplot (v 1.0.2) to show the output of the analysis included a list of enriched biological processes (BP) of GO terms and the genes included in these items.

### RBP motif analysis

Based on the rMATS output results, the complete gene sequences of 24 selected genes, ranging from upstream ES to downstream EE, were extracted using TBtools (75). Multiple sequence alignment was performed using Clustal Omega (https://www.ebi.ac.uk/Tools/msa/clustalo/) with the default settings. RBP motif changes were predicted based on splicing factor binding sites information from RBPmap (https://rbpmap.technion.ac.il/index.html) (76, 77).

### Long-read RNAseq data analysis

ISO-Seq data from the human brain cortex was obtained from the SRA datasets under accessing number PRJNA664117 (human) (39). The datasets included human fetal cortex samples at 14 to 17 wpc and adult cortex samples from individuals aged 24 to 89 years old. To ensure a comprehensive representation of the mouse developmental timeline, we included an additional set of Nanopore long-read sequencing data for mouse NSCs during brain development (SRP321063) (40). For data processing, Pychopper was used to filter and classify reads. Minimap2 was employed to align the processed reads to the reference genomes of human and mouse. Alternative splicing events were analyzed using IGV, which visualized the genomic regions exhibiting selective splicing changes.

### Cell culture and transfection

The SH-SY5Y and N2A cell lines are provided by the Cell Resource Center (IBMS, CAMS/PUMC). The SH-SY5Y cells were cultured in F12/MEM medium supplemented with 15% FBS, while N2A cells were cultured in DMEM medium containing 10% FBS. Both cell lines were maintained at 37 °C with 5% CO_2_. Two hours prior to transfection, the culture medium was replaced. Transfection was performed using Lipofectamine 3000 with three HNRNPH2-targeting siRNA sequences designed specifically for human and mouse. The two siRNAs with the highest knockdown efficiency were selected through qPCR screening. The siRNA sequences were as follows: human, 5′- CUUACGAUCACAGCUAUGU-3′; mouse, 5′-GGUUAUGGUGGUCAGAGCA-3′. After 48 h, cells were harvested, total RNA was extracted (Thermo Fisher Scientific), and reverse-transcribed into cDNA (Takara Biomedical Technology).

### Mouse brain collection

KM precise time point pregnant mice at embryonic day (e) 11.5, e14.5, and e17.5, as well as 4w mice were purchased form Vital River Animal Technology Co, Ltd (Beijing, China) and housed under a 12-h light/dark cycle at 23 ± 2 °C. All animal studies were approved by the Plastic Surgery Hospital, Chinese Academy of Medical Sciences Institutional Review Board. Embryonic mouse cortical tissues were carefully dissected under a dissecting microscope. After three washes with PBS, total RNA was extracted using TRIzol reagent and subsequently reverse transcribed into cDNA. The remaining tissues were homogenized in RIPA lysis buffer (Thermo Fisher Scientific), containing a protease inhibitor mixture (MedChemExpress), and proteins were extracted by centrifugation.

### Neuronal differentiation

The hESCs H9 cell line was cultured in Matrigel-coated plates and maintained in mTeSR1 medium (StemCell Technologies), with passaging performed using dispase (StemCell Technologies). For differentiation, hESCs were dissociated into single cells using Accutase (StemCell Technologies), and then plated onto PLO (Sigma-Aldrich) and laminin (Sigma-Aldrich)-coated plates, where they were differentiated into NPCs using mTeSR1 medium. NPCs were passaged and cultured in the STEMdiff Forebrain Neuron Differentiation Kit, with daily medium changes for 6 days until NPCs adhered and differentiated into forebrain neuron precursors. Accutase was used again to dissociate the cells, and the medium was switched to the STEMdiff Forebrain Neuron Maturation Kit. After 14 days of culture, forebrain neuron marker expression was assessed, and RNA and protein were extracted for subsequent experiments. Immunofluorescence staining using anti-NeuN (1:200, ab177487, Abcam) and anti-PSD95 (1:200, ab192757, Abcam) was performed to evaluate the efficiency of neuronal differentiation.

### PCR, Western blot, and analysis of splicing isoforms

RT-PCR was performed using specific primers for the minigene transcripts of selected d devASGs. PCR products were analyzed by agarose gel electrophoresis to compare the splicing patterns. Quantitative PCR (qPCR) was used to precisely quantify the relative expression levels of specific splicing isoforms. Primers sequences can be found at Table S9.

### General statistics and plots

All plots were done in R (v 4.1.2) as implemented in Rstudio in addition to pattern diagrams and stacked bar chart. The following R packages were used: GenomicAlignments (v 1.30.1), reshape2 (v 1.4.4), ggplot2 (v 3.4.4), Mfuzz (v 2.54.0), pheatmap (v 1.0.12) (78).

## Data availability

All data are available in the main text or the Supplementary Materials. This study includes no data deposited in external repositories.

## Supporting information

This article contains supporting information.

## Conflict of interest

The authors declare that they have no conflicts of interest with the contents of this article.
