# Supplementary material for: Alternative splicing categorizes organ development by stage and reveals unique human splicing variants linked to neuromuscular disorders
Source: J Biol Chem. 2025 Apr 25;301(6):108542. doi: 10.1016/j.jbc.2025.108542 (PMC12152634; doi:10.1016/j.jbc.2025.108542)
Supplement: Supporting information [file mmc1.zip › Supporting information_revised.docx]

**Supporting information**

**Species-specific alternative splicing in brain and heart development reveals unique human splicing variants linked to neuromuscular disorders**

**Chen Li, Fu-xing Gong, Zhigang Yang, Xin Fu, Hang Shi, Xuejian Sun, Xiaorong Zhang, Ran Xiao**

**
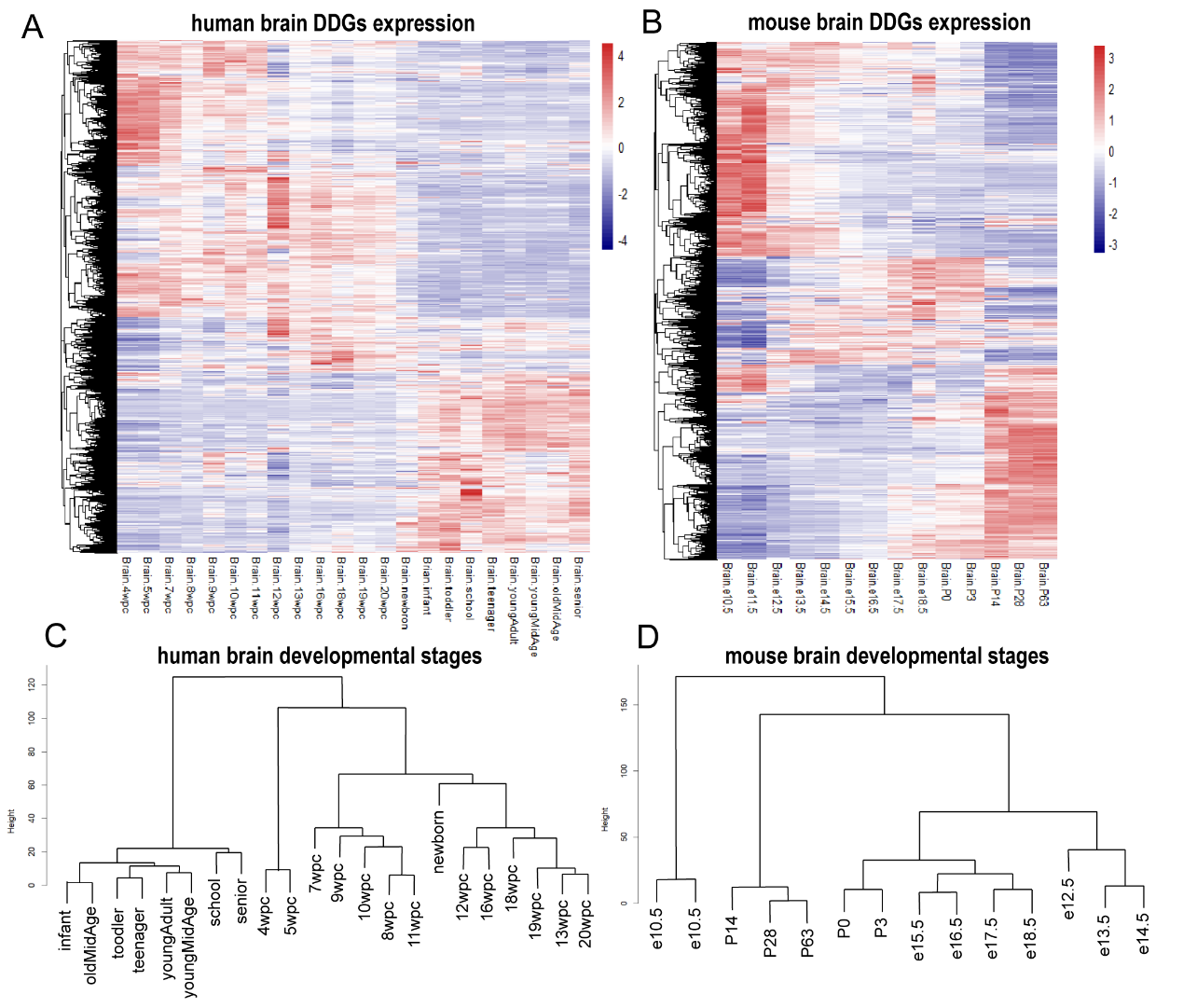
**

**Figure S1. Stage classification of developmentally dynamic genes during brain development.** (A, B) Heatmap of DDGs during human and mouse brain development. (A) Human. (B) Mouse. (C, D) Hierarchical clustering of DDGs during human and mouse brain development. (C) Human. (D) Mouse.

**
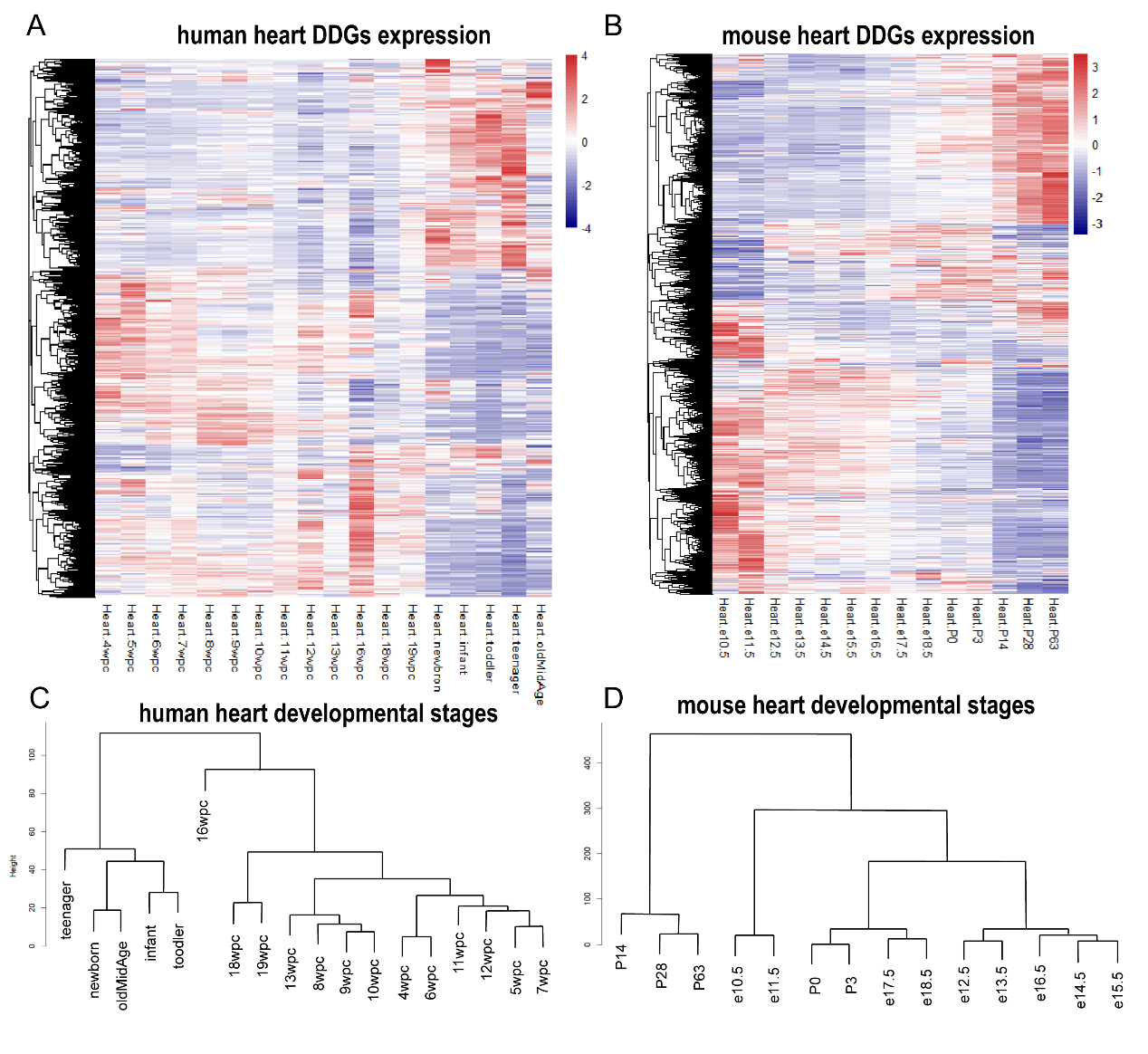
**

**Figure S2. Clustering analysis of differentially expressed genes during heart development.** (A, B) Heatmap of DDGs during human and mouse heart development. (A) Human. (B) Mouse. (C, D) Hierarchical clustering of DDGs during human and mouse heart development. (C) Human. (D) Mouse.


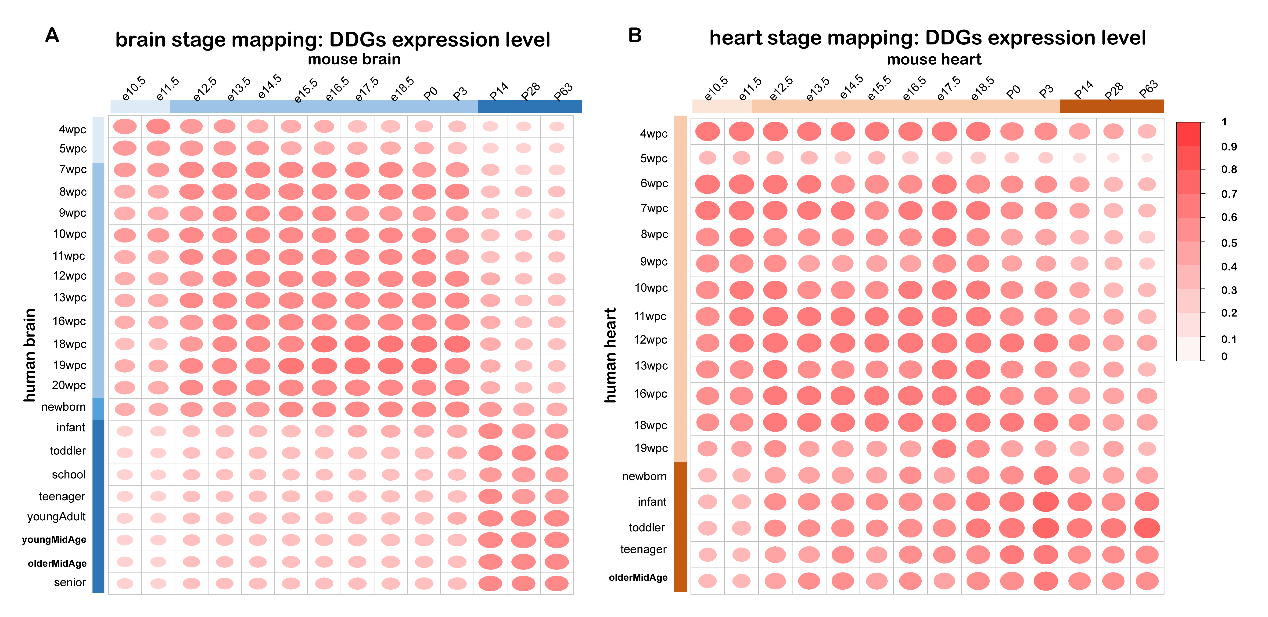


**Figure S3. Correspondences between developmental stages across tissue expression data in human and mouse.** (A) Brain stage mapping using transcription data: The x-axis represents the time points during mouse brain development, while the y-axis represents the time points during human brain development. (B) Heart stage mapping using transcription data: The x-axis represents the time points during mouse heart development, while the y-axis represents the time points during human heart development.


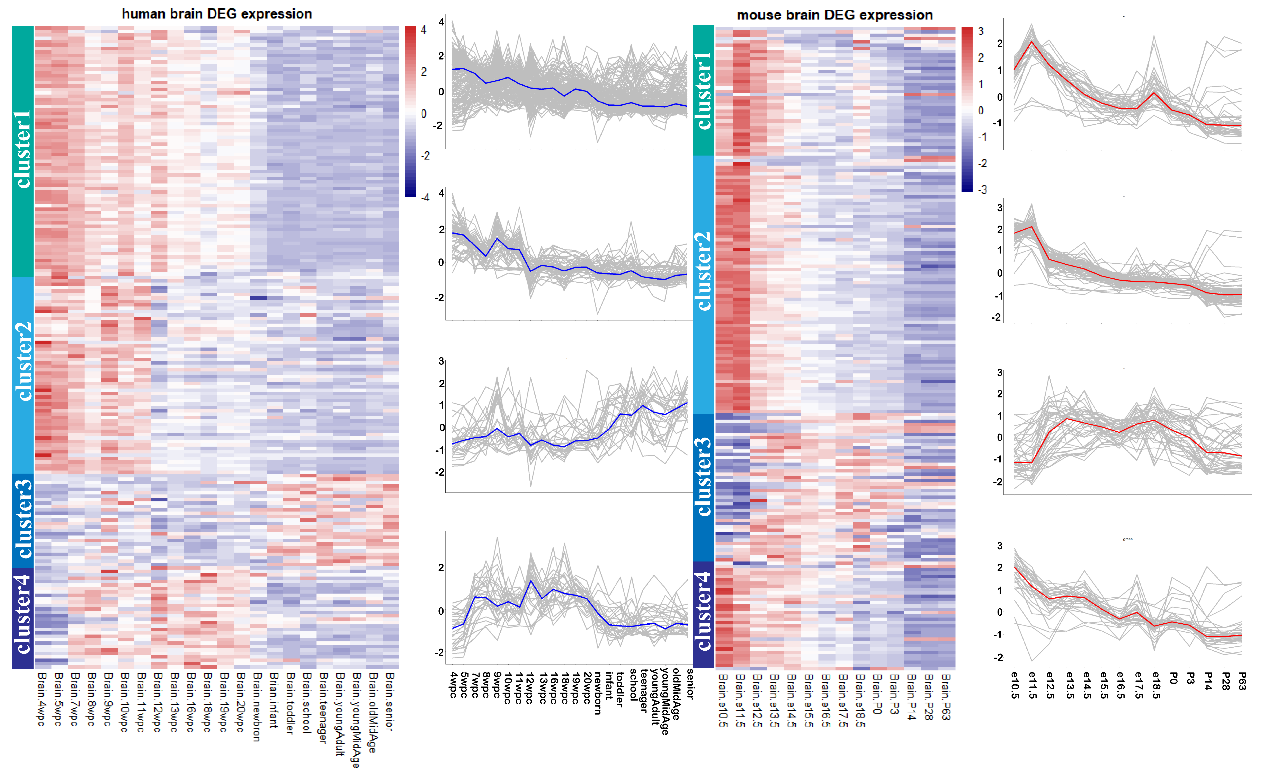


**Figure S4. Clustering analysis and trends of splicing factor expression patterns during brain development.** heatmap illustrates the clustering of SFs expression patterns during human and mouse brain development, with the line plots showing the expression patterns for each cluster. Left: human. Right: mouse.


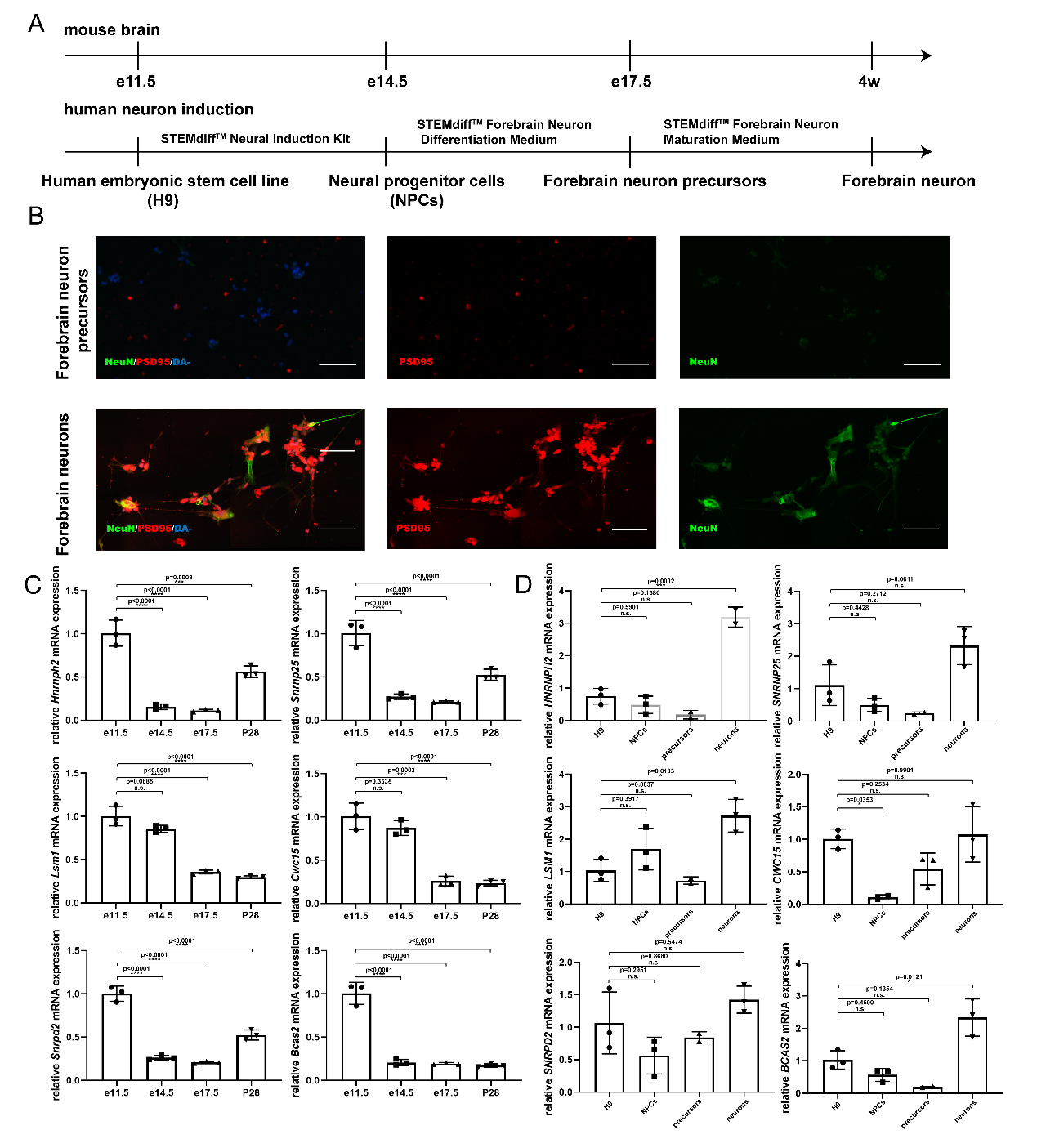


**Figure S5. Expression analysis of human-specific developmentally increasing SFs.** (A) Timeline of mouse brain tissue collection and human forebrain neuron differentiation. (B) Representative images validating neuronal differentiation efficiency. Blue represents neuronal nuclei stained with DAPI, red shows PSD95, a postsynaptic density protein, and green indicates NeuN, a marker for mature neurons. Scale bar 60µm. (C-D) Transcript levels of 6 human-specific upregulated SFs. (C) mouse. (D) human. Each data point represents the mean of three technical replicates. Statistical analysis was performed using one-way ANOVA followed by appropriate multiple comparisons test in GraphPad Prism. Mean ± SD, n= 3 biological replicates, with technical duplicates; * P < 0.05, ** P <0.01, **** P < 0.0.0001.


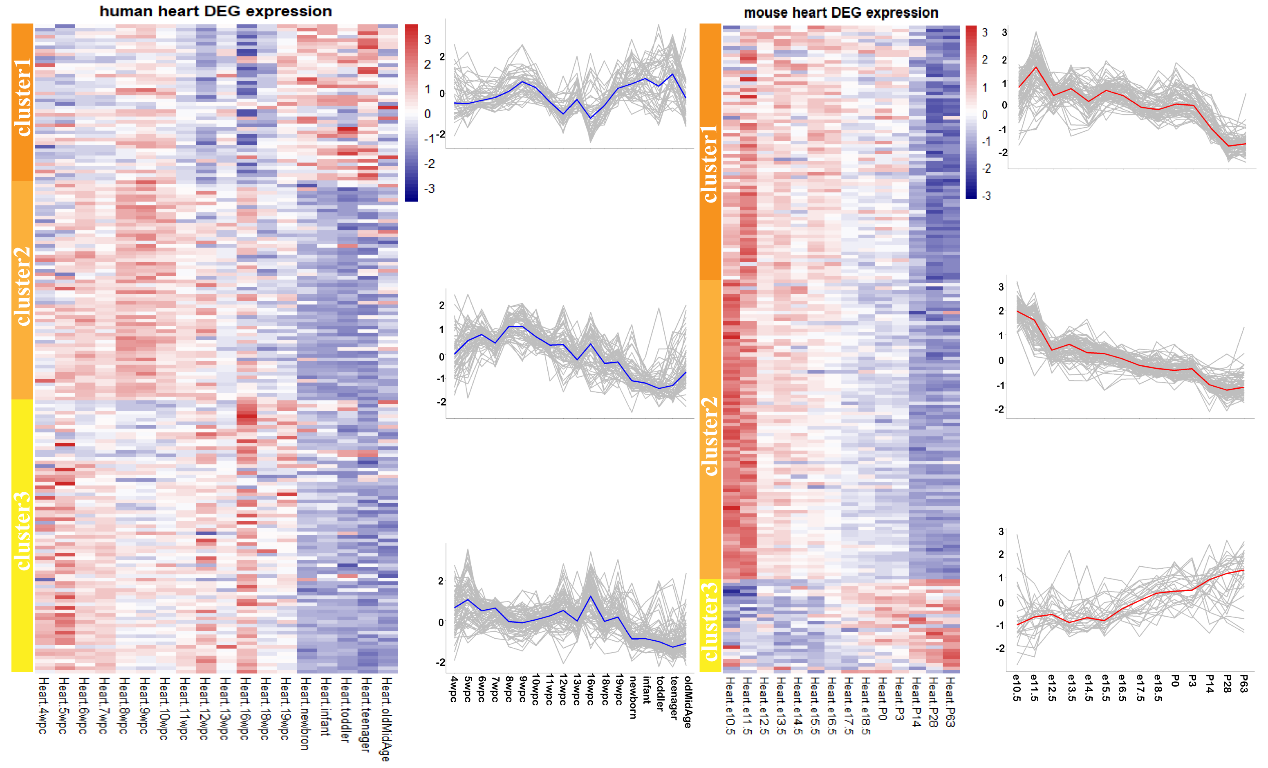


**Figure S6. Clustering analysis of different splicing factor expression patterns during heart development.** heatmap illustrates the clustering of SFs expression patterns during human and mouse heart development, with the line plots showing the expression patterns for each cluster. Left: human. Right: mouse.


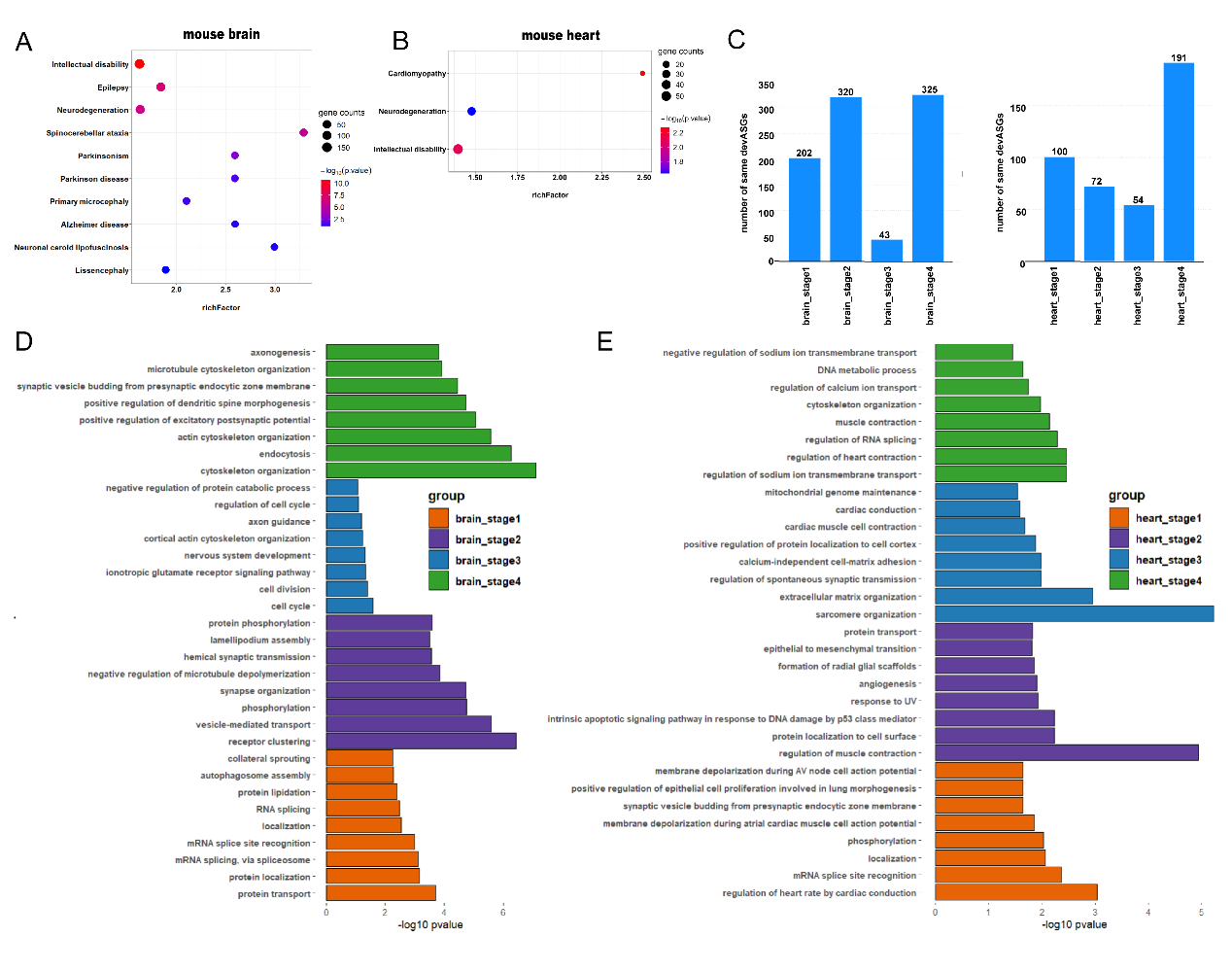


**Figure S7. Diseases enrichment of devASGs during mouse organ development and functional enrichment of genes with conserved splicing changes across species.** (A, B) Diseases enriched by devASGs during mouse brain and heart development (p values < 0.05). (A) Brain. (B) Heart. (C) Number of genes with conserved splicing changes across species during distinct developmental stages of brain and heart. (D, E) GO-BP functional enrichment of genes with conserved splicing changes across species in brain (D) and heart (E).


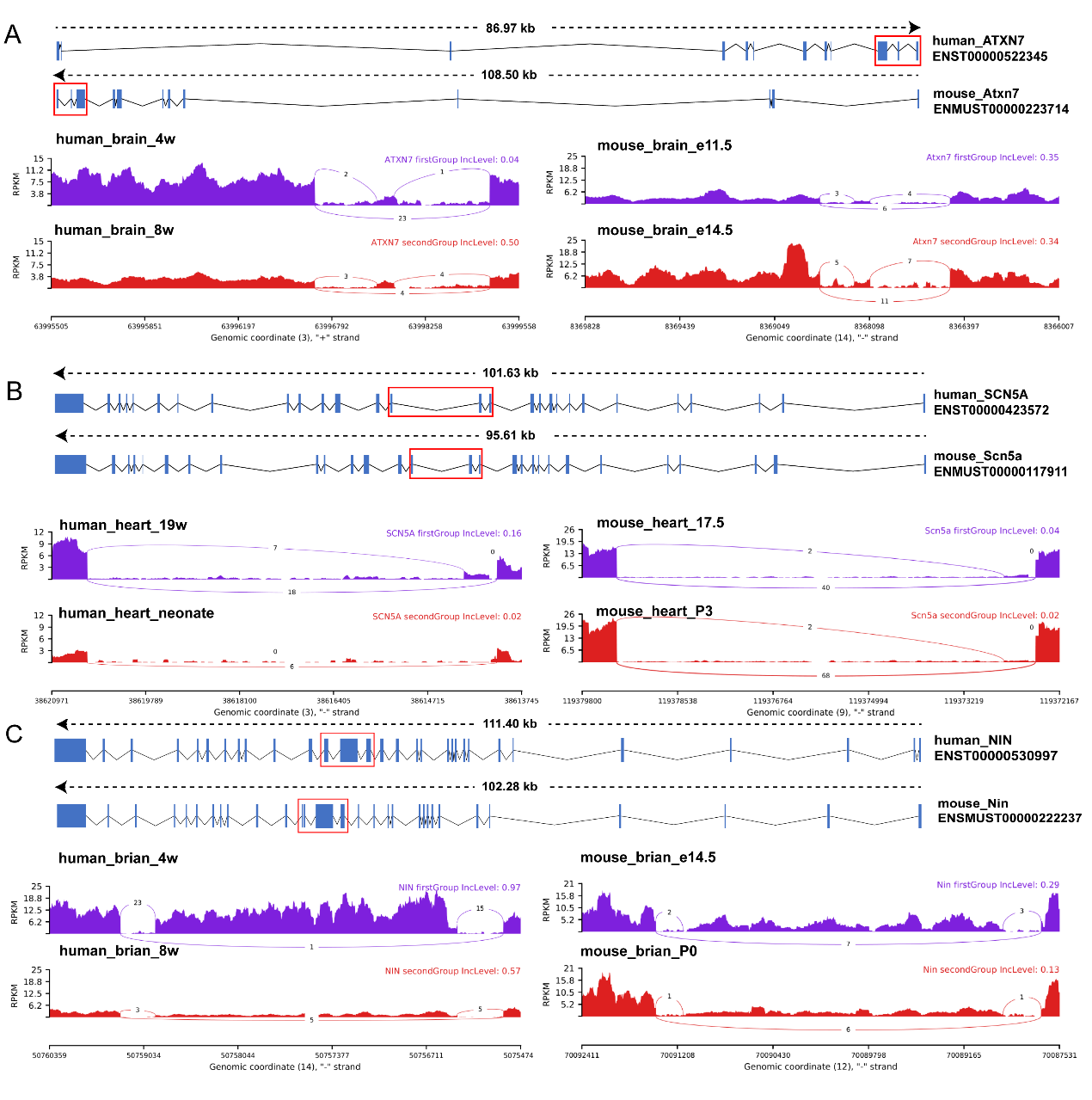


**Figure S8. Different splicing patterns of disease-relative devASGs between human and mouse.** (A) Differential splicing patterns of *ATXN7* in human brain at 4wpc and 8wpc and mouse brain at e11.5d and e14.5d. (B) Differential splicing patterns of *SCN5A* in human heart at 19wpc and neonate and mouse heart at e17.5d and P3d. (C) Differential splicing patterns of *NIN* in human brain at 4wpc and 8wpc and mouse brain at e14.5d and P0d.


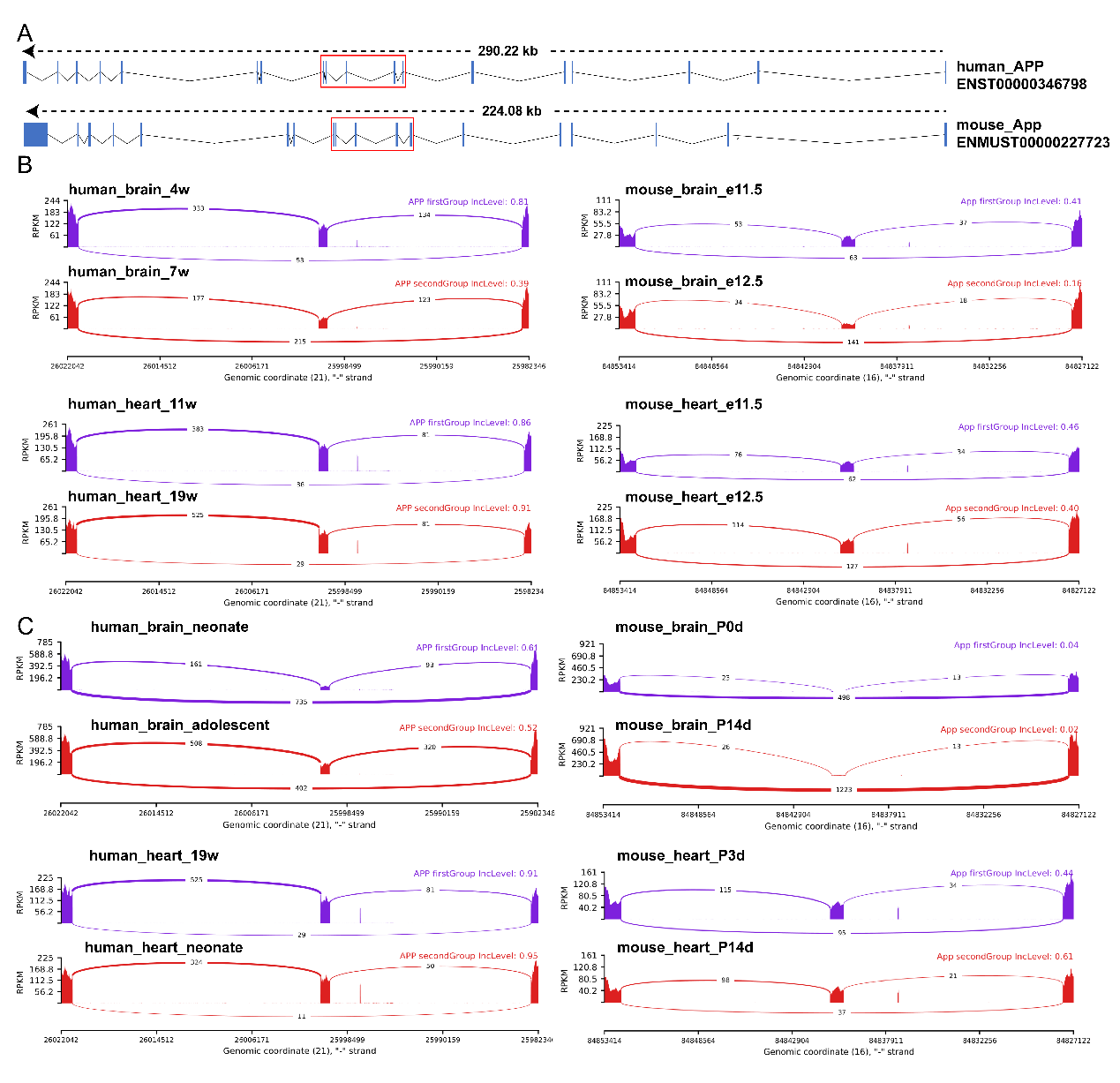


**Figure S9. Differences splicing variations in exon 8 of APP gene between brain and heart development.** (A) Structure of human *APP* genomic locus drawn to scale, with subsequent presentation highlighting the key variant positions. (B) Splicing patterns of exon 8 in the *APP* between brain and heart during early embryonic development. Left: Human. Right: Mouse. (C) Splicing patterns of exon 8 in the *APP* between brain and heart during the postnatal stage. Left: Human. Right: Mouse.


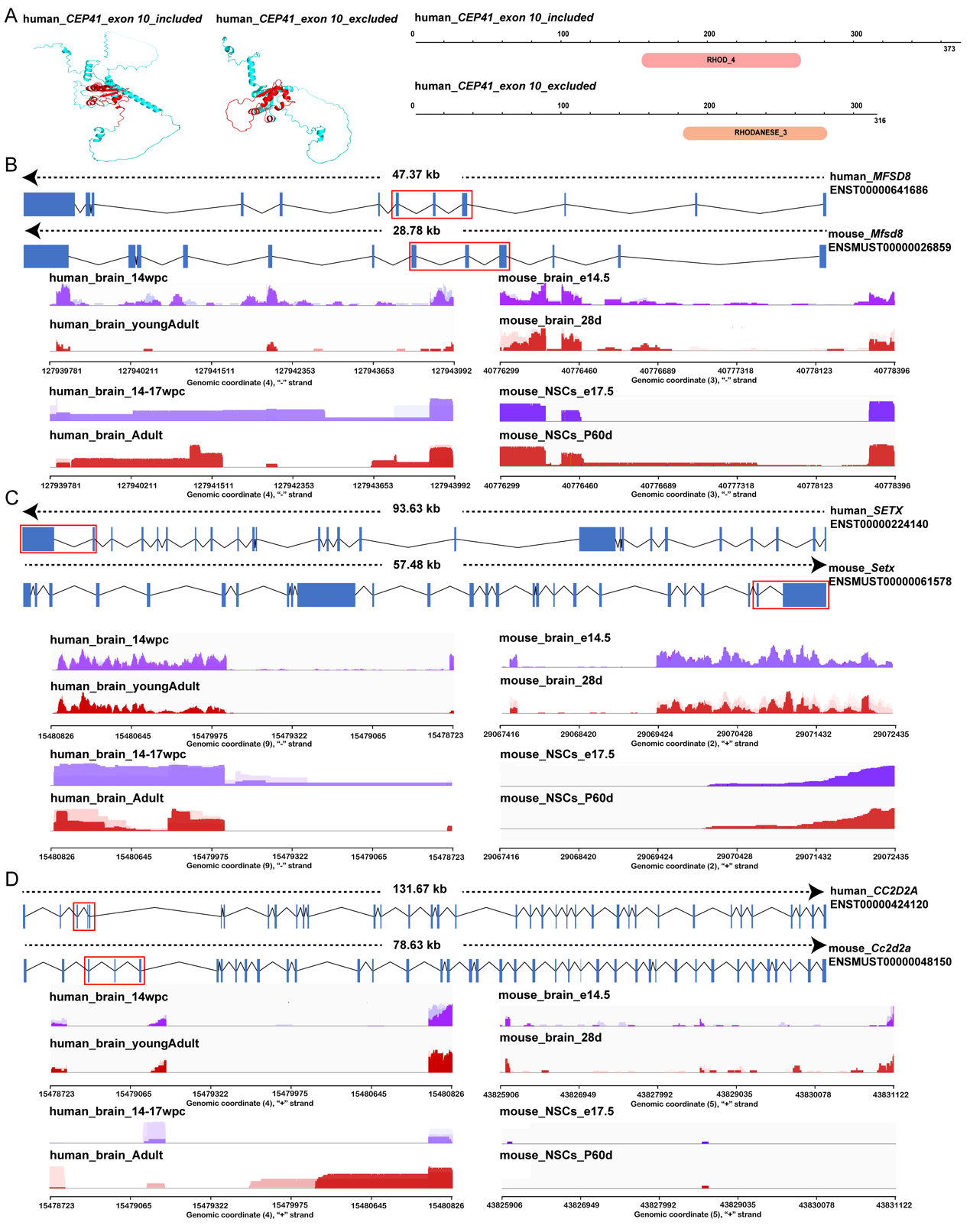


**Figure S10. Different splicing patterns of human-specific devASGs revealed by short-read and long-read sequencing.** (A) Left: AlphaFold-predicted structural models of *CEP41* translation products included and excluded exon 10; Right: InterPro-predicted domain variations in CEP41 with and without exon 10. (B-D) Top: Differential splicing regions in gene structure schematic are highlighted in red box. (B) Middle: Splicing patterns of *MFSD8* based on short-read sequencing data; Bottom: Splicing patterns of *MFSD8* based on long-read sequencing data. (C) Middle: Splicing patterns of *SETX* based on short-read sequencing data; Bottom: Splicing patterns of *SETX* based on long-read sequencing data. (D) Middle: Differential splicing patterns of Middle: Splicing patterns of *CC2D2A* based on short-read sequencing data; Bottom: Splicing patterns of *CC2D2A* based on long-read sequencing data.

**Table S1. Expression matrix of splicing factors, related to Figure 2, 3.**

**Table S2. Results of expression pattern clustering of splicing factors, related to Figure 2,3.**

**Table S3. Fastq data used for splicing events analysis and total devASGs list, related to Figure 4.**

**Table S4. Splicing pattern variations of devASGs across key human brain development stages, related to Figure 4, 6.**

**Table S5. Splicing pattern variations of devASGs across key human heart development stages, related to Figure 4, 6.**

**Table S6. Splicing pattern variations of devASGs across key mouse brain development stages, related to Figure 4, 6.**

**Table S7. Splicing pattern variations of devASGs across key mouse heart development stages, related to Figure 4, 6.**

**Table S8. Expression of human specific neuromuscular disease relative gene during human brain development, related to Figure 5.**

**Table S9. List of primers used for qPCR and PCR analyses.**
